# Recent Advances from the Bench Toward the Bedside in Alzheimer's Disease

**DOI:** 10.1016/j.ebiom.2015.01.002

**Published:** 2015-01-09

**Authors:** Shannon L. Macauley, David M. Holtzman

**Affiliations:** Washington University, Department of Neurology, Hope Center for Neurological Disorders, The Knight Alzheimer's Disease Research Center, 660 S. Euclid Ave., St. Louis, MO 63110, USA

Affecting approximately 30 million people worldwide, Alzheimer's disease (AD) is the most common cause of dementia and there is no cure. The cost to the American public for treating AD is currently estimated at $214 billion per year, a concerning statistic given that the disease population is expected to quadruple by 2050 (Holtzman et al., 2011). By 2030, it is estimated that the global burden of AD will reach $1 trillion USD, which does not take into account the emotional, physical, and financial burden of unpaid caregivers. Although these estimations suggest a bleak horizon for AD patients, caregivers, and their families, great strides in basic research and translational neuroscience in recent years offer new hope for those suffering from this debilitating disease.

There is a unique set of challenges that face researchers when considering how to approach therapeutic intervention for AD. First, the pathological changes that ultimately lead to cognitive decline and dementia begin to accumulate well before symptoms become evident. The two primary pathologies in AD include plaques, extracellular aggregates of the amyloid-beta (Aβ) peptide, and tangles which refer to the intracellular accumulation of aggregated forms of the tau protein. According to the amyloid hypothesis (Hardy and Selkoe, 2002), 10–15 years prior to the onset of dementia, Aβ begins to accumulate within specific regions of the brain and form amyloid plaques. Plaque growth peaks and plateaus just after the onset of clinical symptoms. Aggregated, hyperphosphorylated tau, a marker of neurodegeneration, also begins to accumulate during this pre-symptomatic period. Tau accumulation appears to occur in regions which also have decreased brain volume, synaptic integrity, and glucose metabolism. By the time patients manifest signs of dementia, the pathogenesis of AD is firmly established within the brain and therefore the most effective treatment for AD must not only stop disease progression but must also reverse decades of damage coinciding with Aβ and tau aggregation. Also by the time of diagnosis, Aβ and tau, which appear to be the primary instigators of disease, have initiated a deleterious cascade of secondary disease mechanisms, such as metabolic dysfunction, oxidative stress, and neuroinflammation, which are as equally pernicious to the brain as the primary insult itself. To date, most treatment strategies have tried to target the production or clearance of Aβ or manipulate tau aggregation, while the secondary consequence of plaques and tangles remains largely unaddressed. These approaches have shown limited success in clinical trials to date, most likely due to the timing of therapeutic intervention and the complexity and heterogeneity of disease mechanisms at play in AD. Although targeting plaques and tangles will most likely be integral to any successful treatment strategy for AD, there are other biological processes, such as neuroinflammation, that require equal consideration as researchers move forward toward a holistic and efficacious treatment for AD. Moreover, early intervention, most likely during the pre-symptomatic period is a likely necessity for the most successful treatment of AD.

There have been some important new findings over the last year and a few are highlighted here. One issue that has plagued the AD field is the lack of *in vitro* or *in vivo* systems that mimic all aspects of the human disease. Approximately 20 years ago (Scheuner et al., 1996), several mutations in the genes encoding amyloid precursor protein (APP) and presenilin (PSEN) were discovered to give rise to early-onset familial AD (FAD) through the increased production of the plaque forming peptide, Aβ. Mouse models harboring these human mutations were generated but fail to fully recapitulate human AD. In particular, the overexpression of mutant APP and PSEN1 leads to increased Aβ and plaque formation, yet little to no tau pathology is present in these models. Similarly, models that overexpress mutant forms of human microtubule-associated protein tau display hyperphosphorylated, insoluble tau but lack any appreciable amyloid pathology. Therefore, preclinical studies investigating therapeutic efficacy occurred in models lacking the full breadth of clinical disease, which could be one explanation for why these therapies fail to translate to efficacy in Phase III clinical trials. With the recent publication by Kim, Tanzi, and colleagues in *Nature*, the authors describe the first of its kind *in vitro* model of AD (Choi et al., 2014). By introducing FAD mutations in human neuronal progenitor cells cultured in a three dimensional gelatin matrix, the authors were able to recapitulate both extracellular amyloid plaque pathology as well as intracellular accumulation of aggregated, hyperphosphorylated tau protein, mimicking more closely the pathogenesis of AD in a dish. Not only does this novel 3D culture system provide researchers with a unique opportunity to study the evolution of AD pathology, it provides a tremendous opportunity for high throughout screening of potential drug candidates that can ameliorate both plaque and tangle pathology. Given the investment of time and resources typically involved in drug development and clinical trials, this new technique might offer a more rapid, streamlined approach to candidate selection.

Aside from the importance of producing plaque and tangle pathology in a dish, the findings by Kim, Tanzi, and colleagues also re-emphasize the importance of exploring the genetics underlying AD. Although many FAD mutations in *APP* and *PSEN1* have been well-characterized over the past 20 years, the advent of more sophisticated sequencing techniques allows for the identification of an infinite number of genetic changes associated with AD which could not only give insight into disease pathogenesis but also identify novel therapeutic candidates. Genome-wide association studies (GWAS) have identified at least 20 new loci involved in the increased risk for developing AD (Karch et al., 2014). For example, recent whole-exome and whole-genome wide sequencing strategies identified mutations in the *TREM2* gene as conferring an increased risk for AD by 3.4-fold (Guerreiro et al., 2013, Jonsson et al., 2013). TREM2, or triggering receptor expressed on myeloid cells 2, is a transmembrane protein expressed by myeloid cells, including microglia and peripheral monocytes. Although endogenous ligands for TREM2 remain unknown, TREM2 regulates phagocytosis and the neuroinflammatory response to pathology within the brain. The identification of *TREM2* mutations provides further confirmation that neuroinflammation, specifically microglial activation, is a significant component of AD pathogenesis and is an important component of the comprehensive treatment of AD. Although the role of TREM2 expression on microglial function in the context of Aβ and tau is still up for debate (Ulrich et al., 2014, Melchior et al., 2010, Kleinberger et al., 2014), the importance of GWAS for furthering both the basic understanding of AD as well as the importance in elucidating new therapeutic avenues to pursue is unequivocal.

Tremendous strides in working toward the goal of an Alzheimer's therapeutic have been uncovered by fundamental scientific research. By identifying the timeline of pathological changes in the AD brain, researchers can make more informed decisions on clinical trial design and therapeutic interventions. Development of novel basic science tools, such as the human neural stem-cell-derived 3D culture system, provides an unprecedented new way for studying pathological interactions while simultaneously providing a high throughput screen for possible drug candidates in AD. Using GWAS as a basic method to understand individual risk for developing AD also provides researchers with druggable targets and a more comprehensive understanding of the cascade of disease processes. Taken together, basic and translational scientists are working in concert to bring about changes in the field of AD therapeutics.

## Conflicts of Interest

DMH is a co-founder and serves on the scientific advisory board of C2N Diagnostics, LLC and consults for Genentech, Eli Lilly, AstraZeneca, and Neurophage. His lab receives grants from C2N Diagnostics, Janssen, and Eli Lilly.
